# Differential diagnosis between dilated cardiomyopathy and ischemic cardiomyopathy based on variational mode decomposition and high order spectra analysis

**DOI:** 10.1007/s13755-023-00244-9

**Published:** 2023-09-20

**Authors:** Yuduan Han, Yunyue Zhao, Zhuochen Lin, Zichao Liang, Siyang Chen, Jinxin Zhang

**Affiliations:** 1 Department of Medical Statistics, School of Public Health, Sun Yat-sen University, Guangzhou, China; 2 Department of Cardiology, The Third Affiliated Hospital of Sun Yat‐sen University, Guangzhou, China; 3 Department of Medical Records, The First Affiliated Hospital of Sun Yat-sen University, Guangzhou, China

**Keywords:** Dilated cardiomyopathy, Ischemic cardiomyopathy, Electrocardiogram, Variational mode decomposition, Bispectrum, Classification

## Abstract

The clinical manifestations of ischemic cardiomyopathy (ICM) bear resemblance to dilated cardiomyopathy (DCM). The definitive diagnosis of DCM necessitates the identification of invasive, costly, and contraindicated coronary angiography. Many diagnostic studies of cardiovascular disease have tried modal decomposition based on electrocardiogram (ECG) signals. However, these studies ignored the connection between modes and other fields, thus limiting the interpretability of modes to ECG signals and the classification performance of models. This study proposes a classification algorithm based on variational mode decomposition (VMD) and high order spectra, which decomposes the preprocessed ECG signal and extracts its first five modes obtained through VMD. After that, these modes are estimated for their corresponding bispectrums, and the feature vector is composed of fifteen features including bispectral, frequency, and nonlinear features based on this. Finally, a dataset containing 75 subjects (38 DCM, 37 ICM) is classified and compared using random forest (RF), decision tree, support vector machine, and K-nearest neighbor. The results show that, in comparison to previous approaches, the technique proposed provides a better categorization for DCM and ICM of ECG signals, which delivers 98.21% classification accuracy, 98.22% sensitivity, and 98.19% specificity. And mode 3 always has the best performance among single mode. The proposed computerized framework significantly improves automatic diagnostic performance, which can help relieve the working pressure on doctors, possible economic burden and health threaten.

## Introduction

Electrocardiogram (ECG) is an electrophysiological method for recording the cardiac activity [1]. It has gained wide application in clinical settings owing to its non-invasiveness, affordability, convenient operation, and high time resolution [2]. Dilated cardiomyopathy (DCM) is a non-ischemic myocardial disease with structural or functional myocardial abnormalities [3]. Early diagnosis and treatment of DCM can be significantly helpful to improve the prognosis of patients. But in terms of clinical manifestations, ischemic cardiomyopathy (ICM) is similar to DCM, while the difference in treatment is huge [4]. Explicit exclusion of ICM requires patients to undergo coronary angiography (CAG), an invasive diagnostic technique that entails stringent requirements for surgical instruments and an operating team. Moreover, CAG imposes a considerable economic burden on patients, serving as an additional and unnecessary surgical intervention for individuals with DCM.

Many methods based on gene sequencing and biomarkers have been utilized to assist in the diagnosis of DCM and ICM. Such as plasma metabolomic profiles, expression of Nrf2, syndecan-1, et al. [5–7]. However, the utilization of gene sequencing or uncommon biochemical tests is limited, posing additional financial strain on patients. The laboratory-based approaches, as diagnostic methods for DCM, face challenges in meeting the requirements of routine examinations. Hence, the development of ECG signal analysis techniques is crucial for giving doctors a second opinion on the proper diagnosis of DCM. In this study, we specifically evaluated the diagnostic potential of ECG signals and compared their effectiveness to that of CAG, considered the gold standard.

The ECG analysis methods based on the time–frequency domain, nonlinear domain, and machine learning are increasingly used in DCM detection. In a heart rate variability (HRV) analysis based on ECG signals, HRV parameters such as MeanRR, SDNN, and pNN50 were extracted based on the classification and regression tree algorithm, and included in the produced models, resulting in a classification accuracy of 73.3% [8]. In a spectra analysis method, the quantification of respiratory sinus arrhythmia (RSA) through HRV analysis facilitated the extraction of mean, standard deviation, and nonlinear features. The results showed a significant difference between ICM and DCM subjects (*P* = 0.013) with a sensitivity of 83% and specificity of 90% [9]. In another work, high-resolution joint symbolic dynamics and segmented Poincaré plot analysis were used to ECG signals. They reported a maximum classification accuracy of 84.2% [10]. And in a method based on discrete wavelet transform and K-nearest neighbor (KNN), the highest accuracy between DCM, hypertrophic cardiomyopathy and myocardial infarction reached 96.7% [11].

Variational mode decomposition (VMD), an innovative technique for signal decomposition, was recently introduced by Dragomiretskiy and Zosso [12]. VMD is a modal variational and signal processing technique that is adaptive and entirely non-recursive. The modes obtained through VMD are less susceptible to noise, and it is backed by suitable mathematical modeling. VMD has been used in many biological, voice, and seismic signal processing applications because of its excellent performance in these fields [13]. In the realm of ECG processing, many studies have utilized VMD's ability to capture local variations of clinical components by exploiting the morphological similarities between the mode and the QRS complex [14, 15]. However, there is no unified method for the processing of deconstructed modes. In a sleep apnea detection study based on ECG, the feature vector was constructed by computing spectral entropies, interquartile range, and energy from four modes obtained through VMD. This approach achieved a maximum classification accuracy of 87.5% using KNN [13]. Similarly, a study focused on ventricular arrhythmia recognition employed VMD to extract a total of 24 features, including temporal, spectral, and statistical measures from five modes. The highest accuracy attained was 99.18% [16]. Furthermore, a method based on decision tree (DT) selected hybrid features solely from mode 3 of VMD to discriminate different cardiac arrhythmias, achieving an accuracy of 98.89% [14]. As of yet, no consensus has been reached regarding the optimal number of modes or the subsequent modal deconstruction technique for ECG analysis.

The higher order statistics approach is widely utilized to extract the subtle changes in the biosignals, with bispectrum being a prominent higher order spectra (HOS) parameter [17]. The nonlinear parameters that the second order statistics fail to represent can be extracted by using the bispectrum. And modes of VMD encompass a plethora of information. Based on this, we propose a novel method based on bispectrum and bispectral features for modes obtained through VMD of single-lead ECG signals.

In this paper, a new feature extraction based on VMD and bispectrum, namely bispectral features of the modes obtained through VMD for DCM detection is proposed. The ECG data sets are decomposed into five modes using VMD, followed by bispectral analysis of these modes. And various features including bispectral features, the peak value of PSD, and nonlinear features are calculated from the corresponding bispectral matrix of each mode. For the categorization of ECG signals into DCM and ICM, the characteristics are fed to DT, support vector machine (SVM), KNN and random forest (RF) classifiers, evaluating their performance using assessment indices include sensitivity, specificity, accuracy, and the area under the receiver operating characteristic curve (AUC). Our findings indicate that the RF classifier demonstrates superior performance.

## Methodology

**Fig. 1:**
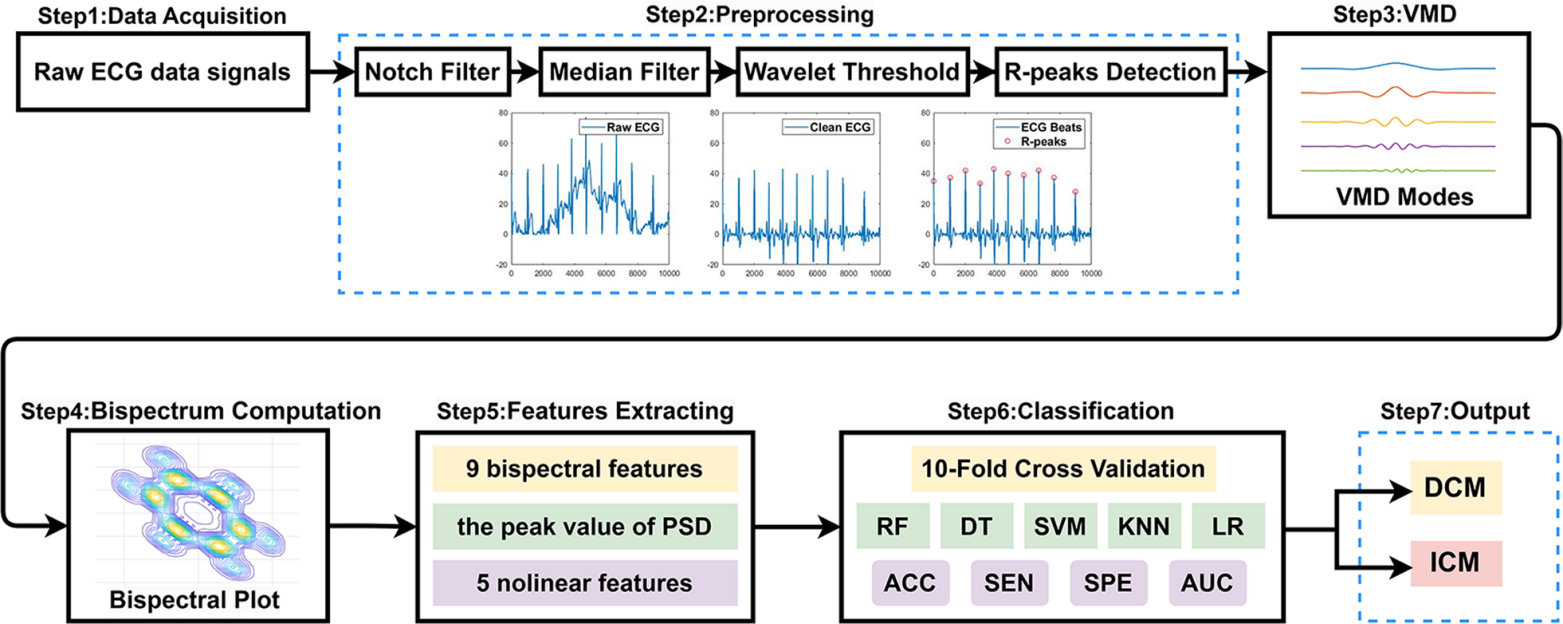
Overview diagram of ECG signals classification using VMD-bispectrum method

The diagram of proposed method is shown in Fig. 1.

### Dataset

In this study, 75 in-patients who were admitted to Sun Yat-sen University's Third Affiliated Hospital over a 5-year period (2006–2021) were retrospectively identified. There were 38 patients diagnosed with DCM and 37 patients diagnosed with ICM among them.

**Fig. 2:**
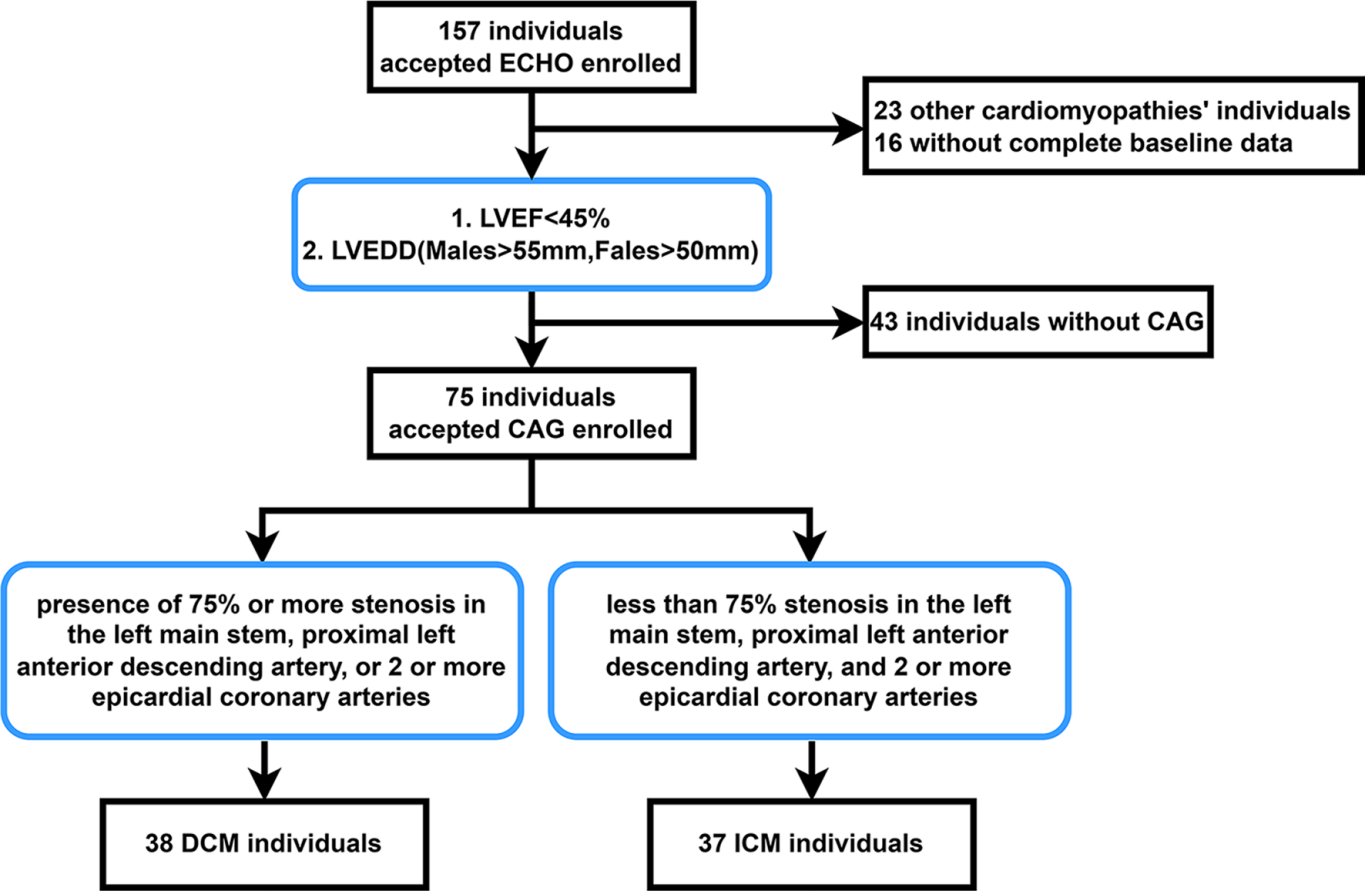
A flow diagram indicating the selection of individuals

To gather data on demographics, co-morbidities, laboratory markers, and ECG signals, a thorough evaluation of electronic medical records was conducted. Figure 2 depicts the flowchart of data collection included in this study. Except for those with additional cardiomyopathies and those without complete baseline data, the echo in both groups showed a dilated left ventricle with an ejection fraction of less than 45%. Then, according to CAG, ICM was defined as having a 75% stenosis in the left main stem, the proximal left anterior descending artery, or two or more epicardial coronary arteries, and DCM as having a stenosis less than that [10].

Table 1 shows baseline characteristics of patients in DCM and ICM, which included demographic, clinical, and echocardiographic characteristics.

**Table 1:** Demographic, clinical, and echocardiographic characteristics of patients with ICM and DCM

Variable	Ischemic cardiomyopathy (*n* = 37)	Dilated cardiomyopathy (*n* = 38)	*P* value
Age	62.35 ± 12.71	63.89 ± 12.65	0.600
Gender (M), *n* (%)	33 (89.2)	24 (63.2)	0.018
Weight (kg)	62.97 ± 9.21	65.11 ± 16.59	0.495
Height (cm)	164.89 ± 9.01	163.79 ± 8.95	0.597
Smoking, *n* (%)	26 (70.3)	14 (36.8)	0.008
Alcohol, *n* (%)	4 (10.8)	7 (18.4)	0.545
Hypertension, *n* (%)	15 (40.5)	14 (36.8)	0.927
Diabetes mellitus, *n* (%)	13 (35.1)	5 (13.2)	0.050
LVEDD (mm)	62.38 ± 6.16	63.76 ± 5.39	0.303
LVEF (%)	33 ± 7	34 ± 8	0.585
Septal thickness (mm)	10.59 ± 1.40	10.34 ± 1.30	0.421

In this investigation, a digital resting surface ECG with a V5 lead was employed. A sample frequency of 1000 Hz was chosen. The program used for signal processing and analysis was MATLAB 2021a. The Human Ethics Boards of the School of Public Health at Sun Yat-sen University gave their approval to the project (2021-No. 081). The Declaration of Helsinki was followed in the execution of the study. Being a retrospective study, informed consent was not necessary.

### Preprocessing

The parser analyzed the XML data containing electrocardiography. To mitigate the influence of power lines interferences, baseline drift and muscle contraction noise presented in the original ECG signals, we apply the 50 Hz notch filter, the median filter, and the wavelet threshold denoising in turn [18]. The ECG signals are fragmented into multiple tiny pieces to ensure data standardization, with each section representing one heartbeat. The Pan-Tompkins algorithm is used to identify the R-peaks. With its straightforward computation and simple implementation, this approach is a reliable R-peak identification.

Each series of 300 samples preceding a QRS peak, 300 samples following the peak, and the QRS peak itself are consolidated into a 601-sample segment, which is subsequently regarded as a single ECG beat for subsequent analysis after detecting the QRS complex. We exclude the initial and final beats from the entire dataset to ensure a consistent count of 601 sample points [19].

### Variational mode decomposition

The VMD approach iteratively decomposes the ECG signal *f* into a *K* set of discrete modes ${u}_{k}$, compactly supports around their center frequencies [20]. The VMD constrained problem is mathematically defined as:

1 $$\begin{aligned} & \mathop {\min }\limits_{{\{ u_{k} \} ,\{ \omega_{k} \} }} \left\{ {\sum\limits_{k = 1}^{K} {\left\| {\partial_{t} \left[ {\left( {\delta (t) + \frac{i}{\pi t}} \right)\;*\;u_{k} (t)} \right]e^{{ - i\omega_{k} t}} } \right\|_{2}^{2} } } \right\} \\ & s.t.\;\sum\limits_{k = 1}^{K} {u_{k} (t) = f} , \\ \end{aligned}$$

where $\left\{{u}_{k}\right\}:=\{{u}_{1},{u}_{2},\ldots,{u}_{K}\}$ and $\left\{{\omega }_{k}\right\}:=\left\{{\omega }_{1},{\omega }_{2},\ldots,{\omega }_{K}\right\}$ reflect shorthand notations for the *k*th mode of the ECG signal and their center frequencies.

To make the problem unrestricted, the formulation includes both a quadratic penalty term and Lagrangian multipliers, $\lambda$:

2 $$\begin{aligned} & L(\left\{ {u_{k} } \right\},\;\left\{ {\omega_{k} } \right\},\;\lambda ) \\ & \quad : = \alpha \sum\limits_{k = 1}^{K} {\left\| {\partial_{t} \left[ {\left( {\delta (t) + \frac{i}{\pi t}} \right)\;*\;u_{k} (t)} \right]e^{{ - i\omega_{k} t}} } \right\|}_{2}^{2} \\ & \quad \quad + \left\| {f(t) - \sum\limits_{k = 1}^{K} {u_{k} (t)} } \right\|_{2}^{2} \\ & \quad \quad + \left\langle {\lambda (t),\;f(t) - \sum\limits_{k = 1}^{K} {u_{k} (t)} } \right\rangle , \\ \end{aligned}$$

where $\delta (.)$ is the Dirac distribution and $\alpha$ is the bandwidth control parameter. The initial minimization issue is resolved using the alternate direction method of multipliers (ADMM) technique. The following formulation captures the obtained modes' frequency domain representation:

3 $$\hat{u}_{n}^{k + 1} (\omega ) = \frac{{\hat{g}(\omega ) - \sum\nolimits_{i \ne n} {\hat{u}_{i} (\omega )} + (\hat{\lambda }(\omega )/2)}}{{1 + 2\alpha (\omega - \omega_{n} )^{2} }}.$$

Similarly, the optimization of $\omega_{n}$ which represents center frequency is defined as follows:

4 $$\omega_{n}^{k + 1} = \frac{{\int_{0}^{\infty } {\omega \left| {\hat{u}_{n} (\omega )} \right|^{2} d\omega } }}{{\int_{0}^{\infty } {\left| {\hat{u}_{n} (\omega )} \right|^{2} d\omega } }}.$$

**Fig. 3:**
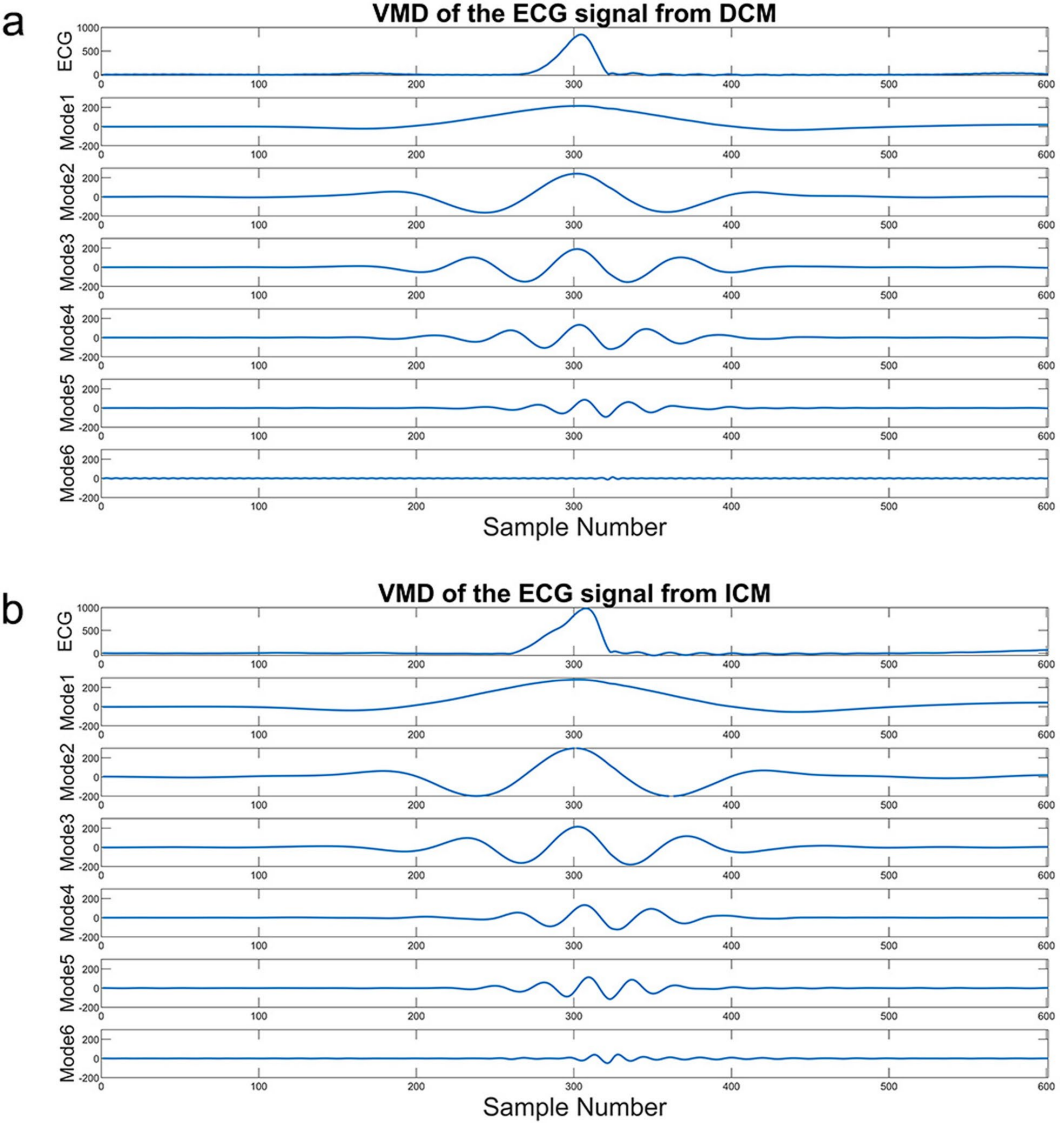
Example six modes of VMD. **a** DCM signal, and **b** ICM signal

The higher modes shoes higher frequency oscillations and contains more energy information about the original signal [16]. Figure 3 shows samples of the six modes based on VMD of ECG signals from two classes of ECG beats. The amplitude values oscillate down significantly from mode 6 onwards and hence contain no significant information, since first five modes are decomposed in this study.

### Bispectrum computation

Following VMD, the modes are estimated using the bispectrum analysis. Higher-order moments or cumulants of a signal can be observed spectrally in higher order spectrum [21]. The third order statistics utilized in this work is referred to as bispectrum.

The power spectra of random process is defined using the Fourier transform of the auto-correlation function. A high-order moment's Fourier transform is how the high-order spectra is described [22]. It is suggested that the HOS can provide precise signal estimation and analysis, and potentially contain richer information compared to low-order spectra.

The bispectrum, which is a two-dimensional function representing the minimal high-order spectra, is a very valuable tool for detecting and quantifying quadratic effects in time series [23].

*x*(*n*) is a stationary, zero-mean, stochastic process with the following definition of the third-order cumulant:

5 $$R_{3x} (\tau_{1} ,\;\tau_{2} ) = E\left[ {x(n)x(n + \tau_{1} )x(n + \tau_{2} )} \right],$$

where ${\tau }_{1}$ and ${\tau }_{2}$ denote the time shift.$E[\cdot ]$ denotes mathematical expectation.

Then, the bispectrum of* x*(*n*) is given by the expression:

6 $$B_{x} (f_{1} ,\;f_{2} ) = \sum\limits_{{\tau_{1} = - \infty }}^{ + \infty } {\sum\limits_{{\tau_{2} = - \infty }}^{ + \infty } {R_{3x} (\tau_{1} ,\;\tau_{2} )} } \cdot e^{{ - j(f_{1} \tau_{1} + f_{2} \tau_{2} )}} ,\quad (\left| {f_{1} } \right|,\;\left| {f_{2} } \right| \le \pi ),$$

where ${f}_{1},{f}_{2}$ are two independent frequencies.

### Feature extraction

This work utilizes nine bispectral features to identify diverse signal qualities, aiming to retain the bispectral information and capture the regularity and irregularity of the signal based on the bispectral matrix [24, 25].

The bispectral features are as follows:

1. Bispectral Brightness: the ratio of the sum of the bispectrum magnitudes above a specific boundary frequency* F* to the sum of all the magnitudes in the bispectrum can be used to express the spectral brightness of a bispectral matrix.
  7 $$F_{1} = \frac{{\sum\nolimits_{i = F}^{\frac{N}{2}} {\sum\nolimits_{j = F}^{\frac{N}{2}} {\left| {\omega (i,\;j)} \right|} } }}{{\sum\nolimits_{i = 0}^{\frac{N}{2}} {\sum\nolimits_{j = 0}^{\frac{N}{2}} {\left| {\omega (i,\;j)} \right|} } }},$$
  where *N* denotes the number of points of bispectral matrix, and $\omega \left(i,j\right)$ is the bispectral amplitude at point $(i,j)$. In this work, we set *F* to 120 Hz.
2. Bispectral Flatness: the degree of closeness between the signal and noise bispectrum is measured by bispectral flatness. It is determined by the geometric mean to arithmetic mean bispectral ratio:
  8 $$F_{2} = \frac{{\sqrt[\frac{N}{2}]{{\sqrt[\frac{N}{2}]{{\Pi_{i = 0}^{\frac{N}{2}} \Pi_{j = 0}^{\frac{N}{2}} \left| {\omega (i,\;j)} \right|}}}}}}{{\frac{1}{N/2}\frac{1}{N/2}\sum\nolimits_{i = 0}^{\frac{N}{2}} {\sum\nolimits_{j = 0}^{\frac{N}{2}} {\left| {\omega (i,\;j)} \right|} } }}.$$
3. Bispectral Roll-off: the frequency that corresponds to the frequency *F* below which a specific proportion of the total bispectral energy is focused is known as the bispectral roll-off. The measure of the signal's non-uniformity around its mean value is the bispectral roll-off. The spectral roll-off is computed as follows,
  9 $$F_{3} = \max F:\sum\limits_{j = 0}^{F} {\sum\limits_{i = 0}^{F} {\left| {\omega (i,\;j)} \right|} \le \beta \cdot } \sum\limits_{j = 0}^{\frac{N}{2}} {\sum\limits_{i = 0}^{\frac{N}{2}} {\left| {\omega (i,\;j)} \right|} } ,$$
  where $\beta$ is the coefficient, which is 0.95 after many experiments in this work.
  And these bispectral entropies have the following formulas:
4. Normalized Bispectral Entropy:
  10 $$F_{4} = - \sum\limits_{i,\;j \in N} {p_{i,j} \log } p_{i,j} ,$$
  where $$p_{i,j} = \frac{{\left| {\omega (i,\;j)} \right|}}{{\sum\nolimits_{i,j \in N} {\left| {\omega (i,\;j)} \right|} }}.$$.
5. Normalized bispectral squared entropy:
  11 $$F_{5} = - \sum\limits_{i,j \in N} {q_{i,j} \log q_{i,j} } ,$$
  where $$q_{i,j} = \frac{{\left| {\omega (i,\;j)} \right|^{2} }}{{\sum\nolimits_{i,j \in N} {\left| {\omega (i,\;j)} \right|^{2} } }}.$$
6. The sum of logarithmic amplitudes of the bispectrum:
  12 $$F_{6} = \sum\limits_{i,j \in N} {\log } (\left| {\omega (i,\;j)} \right|).$$
7. The sum of logarithmic amplitudes of diagonal elements in the bispectrum:
  13 $$F_{7} = \sum\limits_{k \in N} {\log } (\left| {\omega (k,\;k)} \right|).$$
8. The first-order spectral moment of the amplitudes of diagonal elements in the bispectrum:
  14 $$F_{8} = \sum\limits_{k \in N}^{{}} {k\log (\left| {\omega (k,k)} \right|)}$$
9. The second-order spectral moment of the amplitudes of diagonal elements in the bispectrum:
  15 $$F_{9} = \sum\limits_{k \in N} {(k - F_{8} )^{2} \log (\left| {\omega (k,\;k)} \right|).}$$

In addition to the nine bispectral features, we incorporate frequency and nonlinear analysis as complementary measures for each mode. For this purpose, a 16-order autoregressive (AR) model is employed, and the parameters are estimated using Burg's method. The peak value of power spectral density (PSD) in the ECG beats is extracted using the parametric power spectrum estimation method due to its benefit in the analysis of short time series [26]. When the ECG signal is modeled with an AR order of 16, the AR spectrum is served as a reliable alternative to the Fourier spectrum [27].

Furthermore, we acquire the following five features of entropy: approximate entropy, fuzzy entropy, sample entropy, permutation entropy, and complexity [28], expressing modal information intricately. Table 2 provides a comprehensive listing of all 15 features extracted in this study.

**Table 2:** The features range of mean and standard deviation

Mode no		Mode 1		Mode 2		Mode 3		Mode 4		Mode 5	
Signal type		DCM	ICM	DCM	ICM	DCM	ICM	DCM	ICM	DCM	ICM
F1	Mean	0.25744	0.28498	0.70649	0.71847	0.83662	0.81069	0.89847	0.87067	0.63064	0.64305
	Std	0.32293	0.30331	0.20297	0.23306	0.06800	0.08060	0.10716	0.11386	0.08722	0.09524
F2	Mean	0.01176	0.01966	0.00784	0.00732	0.00617	0.00488	0.01382	0.01529	0.08311	0.08823
	Std	0.02620	0.03225	0.01601	0.00971	0.00882	0.00449	0.02385	0.02052	0.04014	0.03780
F3	Mean	113.133	112.975	120.303	120.595	123.121	123.320	124.145	124.036	113.378	112.583
	Std	6.78409	9.13925	3.62911	2.96108	1.55446	1.49324	3.59191	2.90089	6.82796	6.55645
F4	Mean	4.10726	4.12375	3.61804	3.60243	3.32482	3.39483	2.83211	2.92902	3.51764	3.43965
	Std	0.36108	0.35386	0.33452	0.33817	0.22787	0.25354	0.28678	0.35749	0.30880	0.32558
F5	Mean	4.08915	4.10458	3.60051	3.58524	3.30460	3.37154	2.82187	2.91541	3.48190	3.40888
	Std	0.35882	0.35194	0.32879	0.33209	0.22990	0.25345	0.27300	0.34363	0.30684	0.32260
F6	Mean	3.944E+02	1.526E+03	− 1.409E+03	− 1.739E+03	− 1.924E+03	− 9.482E+02	− 2.423E+03	− 2.664E+03	− 2.180E+03	− 2.176E+03
	Std	1.384E+04	1.597E+04	9.846E+03	8.758E+03	8.625E+03	1.174E+04	7.180E+03	5.496E+03	3.518E+03	3.496E+03
F7	Mean	282.616	289.715	130.188	127.491	68.123	77.819	43.139	46.182	85.698	80.464
	Std	209.103	197.708	117.428	70.951	38.062	36.316	9.349	13.643	29.514	28.912
F8	Mean	− 3.097E+05	− 2.931E+05	− 3.577E+05	− 3.624E+05	− 3.816E+05	− 3.746E+05	− 4.038E+05	− 3.965E+05	− 3.028E+05	− 3.020E+05
	Std	9.124E+04	1.022E+05	8.217E+04	7.662E+04	6.200E+04	8.116E+04	6.650E+04	5.941E+04	3.616E+04	3.694E+04
F9	Mean	− 2.814E+14	− 2.552E+14	− 4.052E+14	− 4.131E+14	− 4.591E+14	− 4.500E+14	− 5.458E+14	− 5.133E+14	− 2.245E+14	− 2.232E+14
	Std	1.269E+14	1.299E+14	1.921E+14	1.826E+14	9.343E+13	1.055E+14	1.751E+14	1.804E+14	7.224E+13	7.622E+13
Peak value of PSD	Mean	22.495	14.684	153.507	120.370	474.743	477.037	979.380	707.438	1526.574	1067.572
	Std	59.519	32.098	292.272	293.241	873.091	1110.768	1226.083	1103.626	1966.162	1704.121
Complexity	Mean	0.42979	0.41816	0.30983	0.29731	0.20862	0.20549	0.12104	0.12247	0.06346	0.06421
	Std	0.08621	0.07950	0.06861	0.06395	0.04818	0.05295	0.02216	0.02973	0.00737	0.00770
Sample entropy	Mean	0.15733	0.16265	0.08141	0.09540	0.06222	0.07461	0.05946	0.06802	0.03078	0.03245
	Std	0.14120	0.13244	0.06500	0.06887	0.03998	0.03821	0.01713	0.02498	0.00861	0.01026
Permutation entropy	Mean	0.99950	0.99943	0.99918	0.99922	0.99794	0.99800	0.99506	0.99338	0.98150	0.97971
	Std	0.00082	0.00095	0.00137	0.00128	0.00316	0.00288	0.00747	0.00970	0.02257	0.03075
Fuzzy entropy	Mean	0.19511	0.18553	0.16462	0.16565	0.15211	0.16508	0.17733	0.17885	0.11971	0.10071
	Std	0.08055	0.09376	0.04359	0.04578	0.04393	0.03889	0.03567	0.03498	0.04461	0.05065
Approximate entropy	Mean	0.26822	0.27379	0.21281	0.23085	0.19165	0.21186	0.12930	0.13812	0.04875	0.04917
	Std	0.12007	0.11031	0.07118	0.06722	0.04472	0.04158	0.02166	0.04177	0.00973	0.01244

### Classification

Several machine learning algorithms have been employed to classify cardiac diseases by leveraging the extracted features of ECG signals, encompassing their diverse properties [29]. Utilizing the aforementioned the 15 features, the present study evaluates four classification techniques: SVM, DT, KNN and RF [30–32].

## Results and discussions

In this study, a total of 38 participants diagnosed with DCM and 37 participants diagnosed with ICM collectively provided 6007 ECG beats (3360 DCM beats and 2647 ICM beats). Each ECG beat is decomposed into five modes, from which ECG beat features are extracted. Consequently, a total of 75 features are obtained from each ECG beat. Our research findings indicate that these characteristics can serve as potent predictors.

**Fig. 4:**
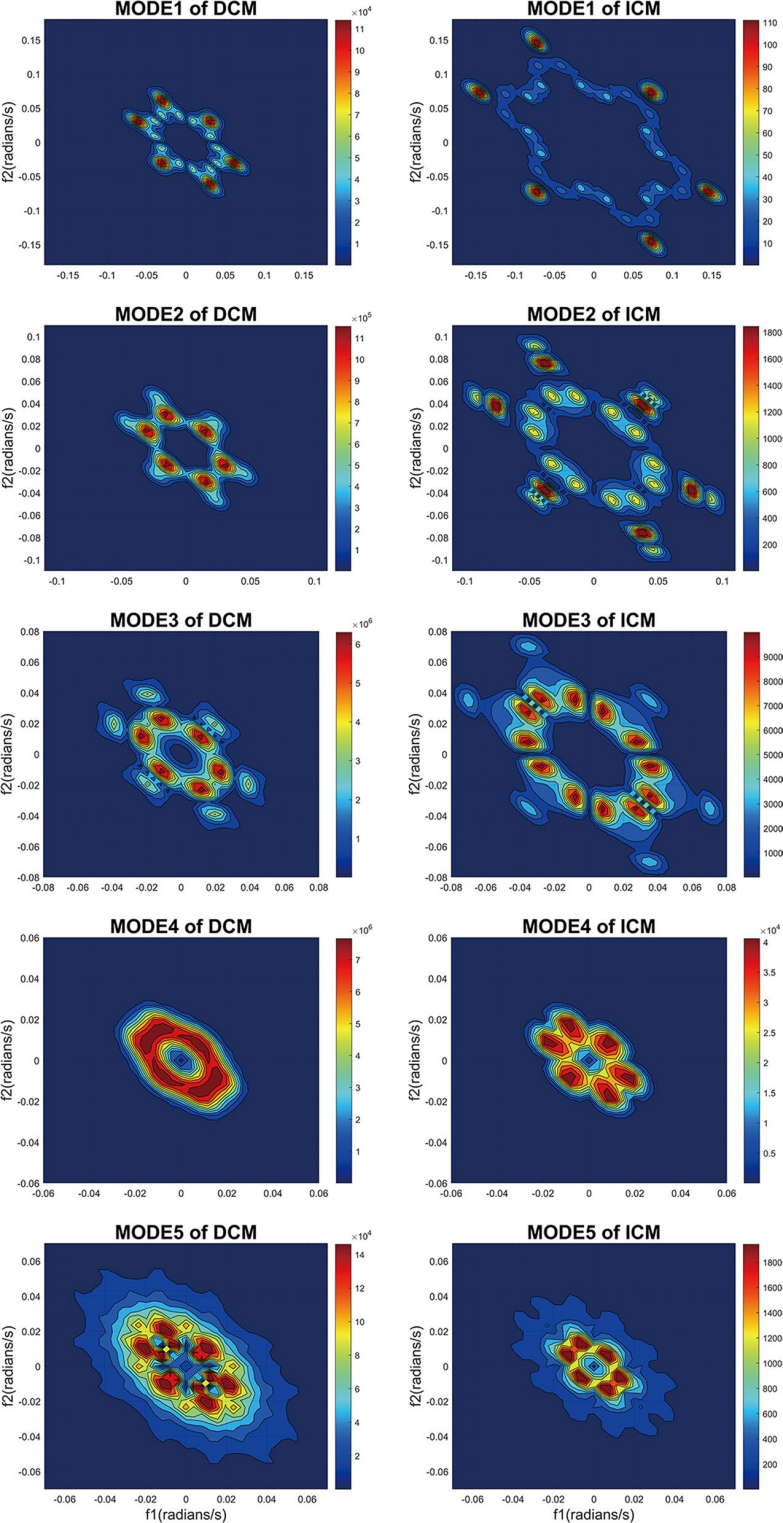
Bispectral contour plots of five modes from two segments in ECG records

Figure 4 depicts the bispectral contour of five modes from a patient of diagnosed with DCM and ICM separately. Each data point in the visualization represents the biamplitude content of the signal at $({f}_{1}, {f}_{2})$, and shows the amount of interaction between frequencies ${f}_{1}$ and ${f}_{2}$. This graphical representation reveals the level of interaction between the mentioned frequencies, which can be attributed to the nonlinear characteristics presented in the ECG signal.

This primary aim of this study is to analyze ECG data using VMD and bispectrum to extract features capable of effectively distinguishing between DCM and ICM. Notably, the oscillation patterns of the five modes obtained from VMD for both DCM and ICM exhibit no discernible differences. This observation can be attributed to the inherent nature of both signals as electrophysiological signals and sharing the same sampling frequency. As a result, comprehensive exploration of the frequency domain information of these modes is pursued, along with the estimation of their respective bispectra to unveil concealed intricacies within the ECG signals.

In the different modes of the bispectrum, the difference can be depicted intuitively. The five modes of DCM exhibit a general resemblance to the corresponding five modes of ICM, albeit with notable dissimilarities in their internal intricacies. Under the condition that the sampling length of the ECG beats are the same, the contour of DCM’s modes appears smoother. Exploiting the characteristics of contour topographic mapping, it becomes evident that DCM exhibits a brighter color. Specifically, the biamplitude content associated with DCM tends to be closer to higher values, whereas the biamplitude content corresponding to ICM tends to be closer to lower values.

The feature vector comprises 15 characteristics for each mode, encompassing 9 bispectral features, the peak value of PSD, and 5 nonlinear features. These characteristics are fed to various classifiers, yielding diverse performance outcomes. In this study, multiple classifiers are employed and their performances are compared to identify the optimal classifier.

Table 2 distinctly illustrates the range of characteristics encompassing the mean and standard deviation of each mode derived from DCM and ICM ECG beats.

In this study, a 10-fold cross validation approach is implemented to mitigate classifier overlap during training and testing. Each time, one equivalent subset of the input data is designated as the testing data, while the remaining subsets are allocated for training purposes. The input data is divided into 10 equal subsets. To ensure rigorous conditions for reporting the results, each subset is employed nine times as training data and only once as test data.

Accuracy (ACC), Sensitivity (SEN), and Specificity (SPE) calculated based on True positive (TP), True negative (TN), False positive (FP), and False negative (FN) are utilized as follows to assess the performance of the suggested method:

16 $$\begin{aligned} ACC & = \frac{TN + TP}{{TN + TP + FN + FP}} \times 100, \\ SEN & = \frac{TP}{{TP + FN}} \times 100, \\ SPE & = \frac{TN}{{TN + FP}} \times 100. \\ \end{aligned}$$

TP represents instances where the input ECG signal is labeled as DCM, and the classifier accurately classifies it within the DCM group. TN refers to cases where the input ECG signal is labeled as ICM, and the classifier correctly assigns it to the ICM group. FP indicates situations where the input ECG signal is labeled as ICM, yet the classifier incorrectly classifies it as DCM. FN denotes cases where the input ECG signal is labeled as DCM, but the classifier erroneously assigns it to the ICM group. We compute the mode classification separately of features, as well as the overall classification across all modes. Given that accuracy provides an intuitive assessment of discriminant effectiveness, it is important to note that high accuracy alone does not guarantee superior classification performance. There is a possibility of encountering a low count of true positives or true negatives [33]. Furthermore, this study computes the AUC value as an evaluation index, which effectively captures the combined impact of sensitivity and specificity.

**Fig. 5:**
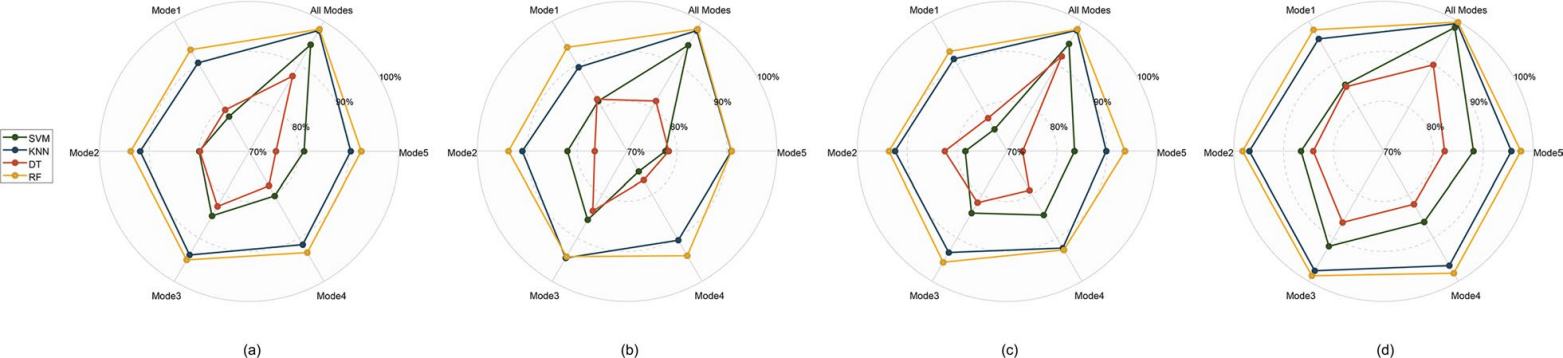
The classification results of various modes,** a** Accuracy, **b** Sensitivity, **c** Specificity, and **d** AUC

Table 3 provides a comprehensive overview of the classification outcomes for the test data, both per mode and across all modes. For each evaluation indicator, the model shows high classification performance and low deviation. The trend of classification results across different modes is illustrated in Fig. 5.

**Table 3:** Performance of four classifiers with various modes

Classifier	Index	All modes	Mode 1	Mode 2	Mode 3	Mode 4	Mode 5
	(%)	(Mean ± std)	(Mean ± std)	(Mean ± std)	(Mean ± std)	(Mean ± std)	(Mean ± std)
RF	ACC	98.21 ± 0.35	93.47 ± 0.96	93.72 ± 1.12	95.02 ± 0.81	93.33 ± 0.86	92.47 ± 0.94
	SEN	98.22 ± 0.69	94.01 ± 2.67	93.76 ± 1.55	94.31 ± 1.82	94.09 ± 2.15	90.95 ± 2.22
	SPE	98.19 ± 0.69	93.04 ± 1.73	93.65 ± 2.39	95.61 ± 2.02	92.75 ± 2.36	93.62 ± 3.05
	AUC	99.81 ± 0.10	98.05 ± 0.48	98.23 ± 0.40	98.71 ± 0.23	98.14 ± 0.29	97.51 ± 0.43
DT	ACC	87.38 ± 1.35	79.57 ± 1.54	79.86 ± 1.42	82.66 ± 1.47	77.92 ± 1.99	75.37 ± 2.82
	SEN	81.58 ± 3.12	82.01 ± 3.80	76.49 ± 3.30	83.73 ± 1.75	76.66 ± 4.14	78.33 ± 6.06
	SPE	91.90 ± 1.66	77.62 ± 3.37	82.48 ± 3.73	81.83 ± 2.90	79.00 ± 4.56	73.12 ± 5.12
	AUC	89.95 ± 1.59	84.92 ± 1.79	84.07 ± 1.26	86.40 ± 1.64	82.19 ± 2.11	82.25 ± 2.27
SVM	ACC	94.64 ± 0.91	78.03 ± 1.97	79.99 ± 1.09	84.86 ± 2.61	80.25 ± 1.93	81.01 ± 1.57
	SEN	94.48 ± 2.58	81.56 ± 5.11	81.95 ± 4.95	85.79 ± 2.94	74.67 ± 4.83	77.68 ± 5.01
	SPE	94.81 ± 2.28	75.05 ± 6.01	78.36 ± 4.56	84.24 ± 5.33	84.70 ± 3.45	83.51 ± 4.21
	AUC	98.57 ± 0.40	85.35 ± 2.13	86.53 ± 0.90	91.90 ± 1.46	86.29 ± 1.79	88.03 ± 1.34
KNN	ACC	97.92 ± 0.61	90.45 ± 1.57	91.82 ± 0.98	93.87 ± 1.37	91.49 ± 1.32	90.35 ± 0.92
	SEN	97.87 ± 1.10	89.39 ± 2.84	90.98 ± 1.79	94.59 ± 1.33	90.50 ± 3.31	90.90 ± 3.00
	SPE	97.94 ± 0.82	91.31 ± 2.67	92.44 ± 1.73	93.34 ± 2.18	92.33 ± 3.16	89.86 ± 2.49
	AUC	99.48 ± 0.27	95.94 ± 1.07	96.86 ± 0.52	97.56 ± 0.67	96.37 ± 0.65	95.61 ± 0.57

Table 4 lists the recording categorization results of the proposed approach with those of several prior studies. The RF has the highest efficiency in mode classification, achieving impressive accuracy, sensitivity, specificity, and AUC value of 98.21%, 98.22%, 98.19%, and 99.81% respectively. The classification performance is better than that of the recent studies on the identification of DCM and ICM. Moreover, within a similar research context, the proposed strategy exhibits comparable classification effectiveness to studies distinguishing DCM from hypertrophic cardiomyopathy through deep learning methods [34], underscoring the advanced nature of the proposed approach.

**Table 4:** Per-recording classification performance (%) comparison of the proposed approach with some recent studies

Authors	Methods and classifiers	Accuracy	Sensitivity	Specificity
Accardo et al. [8]	Linear and non-linear HRV parameters, CART	73.3	–	–
Rodriguez et al. [35 ]	Coupling analysis, SVM	84.2	76.9	88
Rodriguez et al. [36]	HRV of ECG, SVM	92.7	94.1	91.7
Rodriguez et al. [37]	Recurrence plot, SVM	92.3	95.8	86.6
Rodriguez et al. [38]	Pulse transit time of ECG, SVM	89.6	78.5	100
Gunukula et al. [39]	QRS and other statistical characteristics, SVM	76.3	–	–
Adam et al. [11]	Discrete Wavelet Transform features, KNN	96.7	99.6	99.4

There is no predefined criterion for selecting the most suitable classifiers for diverse challenges. Despite the classification performance of all modes is the best, this study evaluates the classification performance individually for each mode. Nevertheless, the four classifiers in this study show a remarkable level of coherence in terms of the classification performance for individual mode. Mode 3 consistently achieve the highest accuracy among the five modes, aligning with the conclusion that it bears the closest resemblance to the QRS complex [14].

The approach employed in this work focuses on the comprehensive exploration of the modal frequency domain following VMD, thereby mitigating the frequency domain incompleteness in the subsequent analysis of various modes. In practical application, this model could diagnose DCM and ICM conveniently without consuming long operation time and financial resources. The analysis of the optimal number of modes obtained from VMD and features adaptation is the direction of our follow-up work.

## Conclusion

We propose a VMD-Bispectrum based approach for distinguishing DCM and ICM. A novel method is put forward to select the bispectral features. The ECG signals are partitioned into individual ECG beats, subsequently decomposed into five modes using VMD. Bispectrum estimation is conducted for each mode. Nine bispectral features corresponding to these modes are recorded, including the peak value of PSD and five entropies. These features are made available to the classifiers for categorizing. The suggested methodology efficiently distinguishes between DCM and ICM of ECG beats, according to experimental data. The RF classifier achieves a classification accuracy of 98.21%, with classification accuracy for each mode being more than 90%. Hence, the suggested methodology's merits include both robustness and universality. In our future work, we intend to apply the proposed methodology to other cardiovascular and cerebrovascular diseases to verify universality, and further improve the performance of our method.
